# Keratocyte loss in corneal infection through apoptosis: a histologic study of 59 cases

**DOI:** 10.1186/1471-2415-4-16

**Published:** 2004-12-24

**Authors:** Geeta K Vemuganti, Kishore Reddy, Ghazala Iftekhar, Prashant Garg, Savitri Sharma

**Affiliations:** 1 Ophthalmic Pathology Service, Brien Holden Eye Research centre, Hyderabad Eye Research Foundation, L.V. Prasad Eye Institute, Hyderabad; 2 Cornea Service, Brien Holden Eye Research centre, Hyderabad Eye Research Foundation, L.V. Prasad Eye Institute, Hyderabad; 3 Jhaveri Micrbiology Centre, Brien Holden Eye Research centre, Hyderabad Eye Research Foundation, L.V. Prasad Eye Institute, Hyderabad

**Keywords:** infectious keratitis, apoptosis, keratocytes, TUNEL stain

## Abstract

**Background:**

Keratocyte loss by apoptosis following epithelial debridement is a well-recognized entity. In a study of corneal buttons obtained from patients of corneal ulcer undergoing therapeutic keratoplasty, we observed loss of keratocytes in the normal appearing corneal stroma, surrounding the zone of inflammation. Based on these observations, we hypothesized that the cell loss in the inflammatory free zone of corneal stroma is by apoptosis that could possibly be a non-specific host response, independent of the nature of infectious agent.

**Methods:**

To test our hypothesis, in this study, we performed Terminal deoxyribonucleotidyl transferase-mediated d-Uridine 5" triphosphate Nick End Labelling (TUNEL) staining on 59 corneal buttons from patients diagnosed as bacterial, fungal, viral and *Acanthamoeba *keratitis. The corneal sections were reviewed for morphologic changes in the epithelium, stroma, type, degree and depth of inflammation, loss of keratocytes in the surrounding stroma (posterior or peripheral). TUNEL positivity was evaluated in the corneal sections, both in the zone of inflammation as well as the surrounding stroma. A correlation was attempted between the keratocyte loss, histologic, microbiologic and clinical features.

**Results:**

The corneal tissues were from 59 patients aged between 16 years and 85 years (mean 46 years) and included fungal (22), viral (15), bacterial (14) and *Acanthamoeba *(8) keratitis. The morphological changes in corneal tissues noted were: epithelial ulceration (52, 88.1%), destruction of Bowman's layer (58, 99%), mild to moderate (28; 47.5%) to severe inflammation (31; 52.5%). Morphologic evidence of disappearance or reduced number of keratocytic nuclei in the corneal stroma was noted in 49 (83%) cases; while the TUNEL positive brown cells were identified in all cases 53/54 (98%), including cases of fungal (19), bacterial (14), viral (13), and *Acanthamoeba *keratitis. TUNEL staining was located mostly in the deeper stroma and in few cases the peripheral stroma. TUNEL positivity was also noted with the polymorphonuclear infiltrates and in few epithelial cells (10 of 59, 17%) cases, more with viral infections (6/10; 60%).

**Conclusions:**

We report apoptotic cell death of keratocytes in the corneal stroma in infectious keratitis, a phenomenon independent of type of infectious agent. The inflammatory cells in the zone of inflammation also show evidence of apoptotic cell death. It could be speculated that the infective process possibly triggers keratocyte loss of the surrounding stroma by apoptosis, which could possibly be a protective phenomenon. It also suggests that necrotic cell death and apoptotic cell deaths could occur simultaneously in infective conditions of the cornea.

## Background

Infections of the cornea are potentially blinding diseases and may be caused by fungi, bacteria, viruses and a few protozoans. Despite best efforts with early diagnosis and specific antimicrobial treatment about one-third of cases require surgical intervention [1]. Those which respond to medical treatment result in scarring which may lead to varying degree of visual disability. The tissue destruction in infectious keratitis, irrespective of the etiologic agent, is the compound effect of cytokines and inflammatory mediators released by the microorganism, host inflammatory cells and the metalloproteases that act on the collagen [2-5]. The aim of treating corneal infections is not only to control the infection but also to restrict the tissue damage so that the corneal stroma maintains the transparency thus retaining its visual function. Though wound healing following surgical procedures including routine keratoplasty and refractive surgeries is well documented [6-11], healing after an infectious etiology is not well documented. We had observed the loss of keratocytes in deeper stroma in *Acanthamoeba *keratitis and had proposed that one of the mechanisms for this loss was apoptosis of keratocytes [12]. We have since then observed the paucity of stromal keratocytes in the zone surrounding the infected area in all types of corneal infections, prompting us to speculate that it is possibly a host mediated response to an infectious process.

Apoptosis is an active process orchestrated by a series of events starting from activation of genes, release of enzymes, specific morphologic changes and ultimately leading to "an environmental-friendly cell death" whereby the dead cells are removed without affecting the bystander cells [13-15]. This is in contrast to necrotic cell death whereby the cell dies with release of various molecules that destroy the surrounding cells and stroma. Though tissue necrosis is an accepted fate of tissues in an infective process, it is associated with unwanted loss of visual function in infections of cornea. Apoptotic cell loss may be an attempt by the host stroma to escape necrosis, thereby minimizing tissue damage.

The corneal buttons removed during therapeutic keratoplasty for uncontrolled infections could possibly throw some light on these events. This study was designed to look for the evidence of apoptotic cell death of keratocytes, along with the morphological changes in corneal tissues in infective conditions.

## Methods

### Patients and samples

Corneal tissues from patients diagnosed as infectious keratitis undergoing therapeutic keratoplasty between 1998 and 2002 were reviewed. The corneal sections of those cases, in which the normal stroma surrounding the zone of inflammation and necrosis was visible, were included in the study. We included 59 patients diagnosed for fungal (22), viral (15), bacterial (14) and *Acanthamoeba *(8) keratitis in the study. The diagnosis of fungal, bacterial and *Acanthamoeba *keratitis was based on the microbiological investigations as described below. The etiologic diagnosis was consistent with histopathology of the corneal buttons excised during therapeutic penetrating keratoplasty. Clinical information was available in 43 cases from the ulcer database of our institute. The cases with missing clinical data on a specific parameter were excluded while calculating the statistical significance of that parameter. The clinical data was analyzed for duration of symptoms, size of ulcer, location, depth of infiltrates, presence of perforation, thinning, anterior chamber infiltration and duration of treatment.

### Microbiology

All patients had undergone microbiological investigations by processing of their corneal scrapings for bacteria, fungus, *Acanthamoeba*. Corneal scrapings were subjected to Grams, Giemsa and Calcofluor white stain for direct smear examination and culture on blood agar, chocolate agar, brain heart infusion broth, thioglycollate broth, Sabouraud's dextrose agar and non-nutrient agar with *Escherichia coli *overlay. Immunofluorescent assay for HSV-1 was done on cold acetone fixed corneal scraping smears using anti-rabbit HSV-1 polyclonal antibody and FITC conjugated swine antirabbit immunoglobulin (DAKO, Denmark). In addition, polymerase chain reaction using primers specific for HSV-1 glycoprotein D gene was done on DNA extracted from corneal scraping with commercially available DNA zol solution (Helena Bio Sciences, UK)

### Histopathology

The excised corneal buttons of the 59 patients were subjected to routine histopathology processing. The paraffin embedded sections were stained with hematoxylin eosin, periodic acid Schiff's and special stains like Gomori's methenamine silver stain and Grams stains. The sections were studied for histologic changes viz. ulceration, inflammation, vascular channels, necrosis, keratocyte loss and other details. Inflammation was graded in a semi-quantitative way as mild, moderate or severe based on the density of the inflammatory cells and the visibility of the stromal collagen in the background. We looked for the absence or the reduced number of the keratocytic nuclei in the corneal stroma surrounding the zone of inflammation.

### TUNEL staining

**T**erminal deoxyribonucleotidyl transferase-mediated d-**U**ridine 5" triphosphate **N**ick **E**nd **L**abeling (TUNEL) staining was performed on all the 59 sections, as per the manufacture's instructions using In-Situ Cell Death Detection Kit, POD (Roche Diagnostics, Manheim, Germany). In brief, the sections were de-paraffinized, re-hydrated and incubated with proteinase K (20 ug/ml) for 10 minutes at room temperature. After rinsing with phosphate buffered saline (PBS) the sections were blocked with 3% hydrogen peroxide in methanol for 10 minutes. The sections were then incubated in the permeabilization solution consisting of 0.1% triton × 100 in 0.1% sodium citrate for 20 minutes. After washing the sections were incubated with TUNEL reaction mix at 37°C in a humidified chamber for 2 hours followed by washing and incubation with POD converter for 2 hours, the sections were stained with diaminobenzidine and counter stained with hematoxylin and observed under the microscope. Sections from the normal corneal tissues obtained from the eyebank corneas were used as control slides. Statistical analysis was done using Chi-Square test and Fisher Exact test.

## Results

We studied the excised corneal buttons of 59 patients who underwent therapeutic penetrating keratoplasty for fungal, bacterial, viral and *Acanthamoeba *keratitis. The mean age of the patients was 46 years (range 15–85 years) with male to female ratio of 1 (22): 1.7 (37). Based on microbiologic and histologic findings, the buttons were diagnosed as fungal keratitis in 22; bacterial in 14, viral in 15 and *Acanthamoeba *keratitis in 8 cases.

### Clinical profile

The clinical features of 43 patients at the time of presentation are given in Table 1. The size and location of the ulcer with depth of infiltrates was noted. Based on the microbiology report, these patients were treated with specific antimicrobial therapy. The cases with advanced disease at presentation or not responding to treatment were subjected to penetrating keratoplasty. The patients were treated for a median duration of 8 days (1–164 days) with specific anti-microbial therapy respectively.

### Histopathology

**Figure 1:**
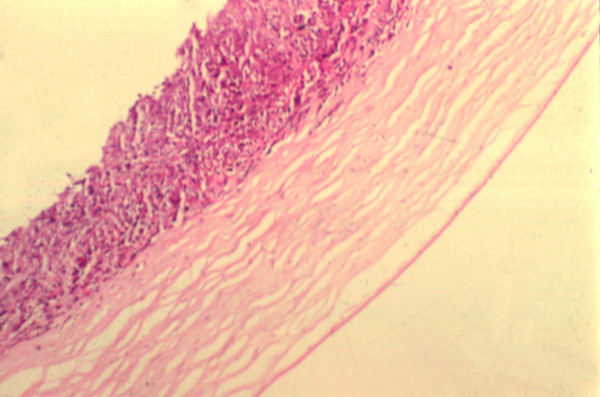
Section of the corneal button from a case of bacterial keratitis showing epithelial ulceration. Note the dense the inflammatory infiltrates in the anterior two-thirds of corneal stroma and the quiet deep corneal stroma. (Hematoxylin and eosin, ×125)

**Figure 2:**
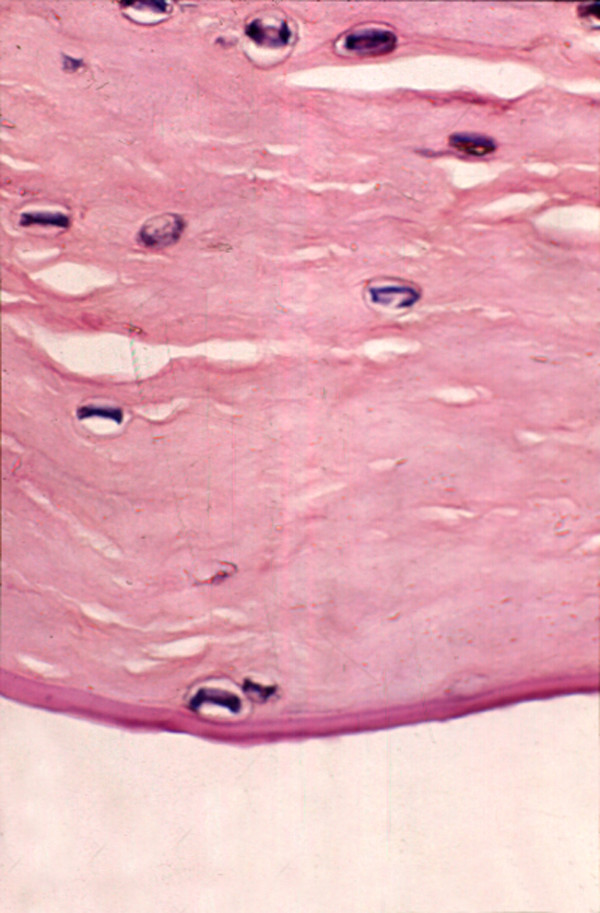
The section from a case of *Acanthamoeba *keratitis showing cysts of *Acanthamoeba *in the deep stroma. Note the complete absence of inflammatory cells in this area as well as the loss of keratocytic nuclei. (Hematoxylin and eosin, ×500)

The histologic changes were observed in the sections of the excised corneal tissues are described in Table 2. The common observations were ulceration (52, 88.1%) (figure 1), destruction of Bowman's layer (58,98%), mild to moderate (28; 47.5%) to severe inflammation (31; 52.5%). Vascular channels were seen in 13.6% of the cases, while necrosis was noted in 48 (81.4%) cases. The morphologic evidence of disappearance or reduced number of keratocytic nuclei (figure 2) in the corneal stroma was noted in 49 (83%) cases. Necrosis was more in cases of fungal keratitis as compared to other infections (22 of 22), while it was minimal in viral infections (7 of 15). Similarly severe inflammation was more in fungal keratitis (16 of 22 cases).

### TUNEL assay

**Figure 3:**
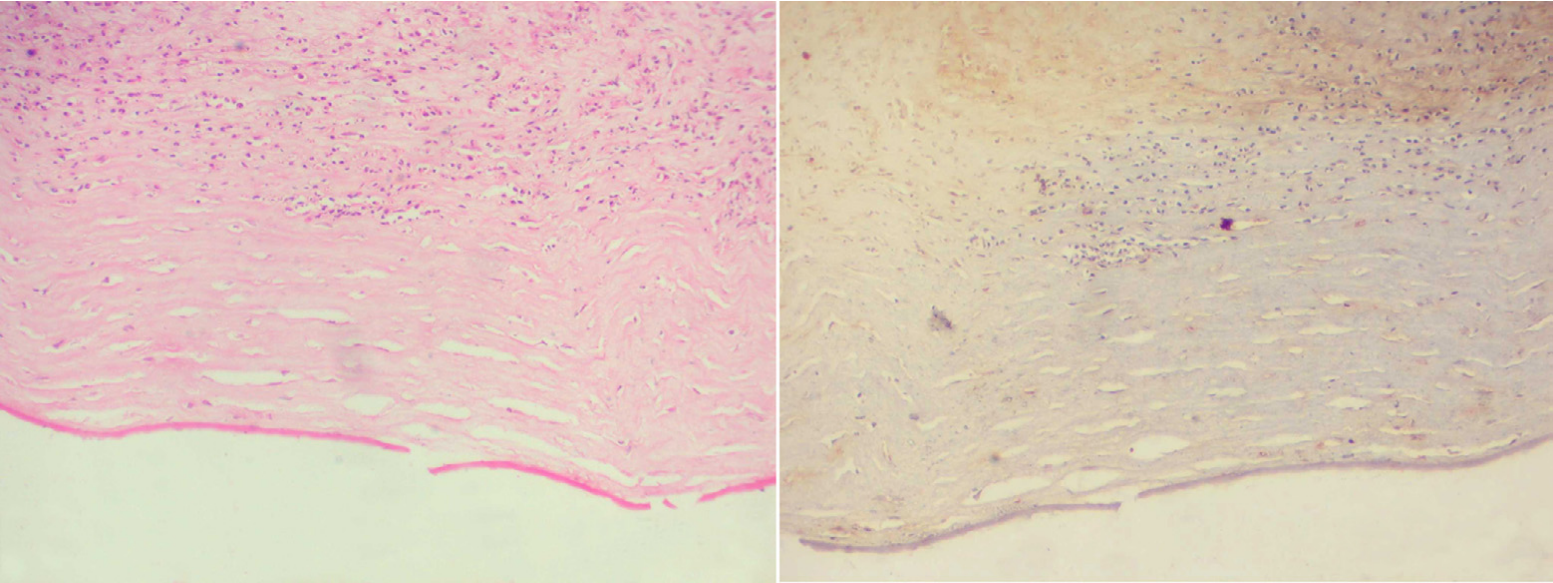
A low power picture of corneal button with Acanthamoeba keratitis showing inflammation in the superficial layers with few keratocytes in the deeper stroma. (Hematoxylin and Eosin, ×100) [Left]. TUNEL stained sections of the same case show dense diffuse brown staining in the inflammatory region in the anterior stroma while the deeper stroma shows brown positivity in the few remaining keratocytic nuclei. (TUNEL ×100) [Right]

**Figure 4:**
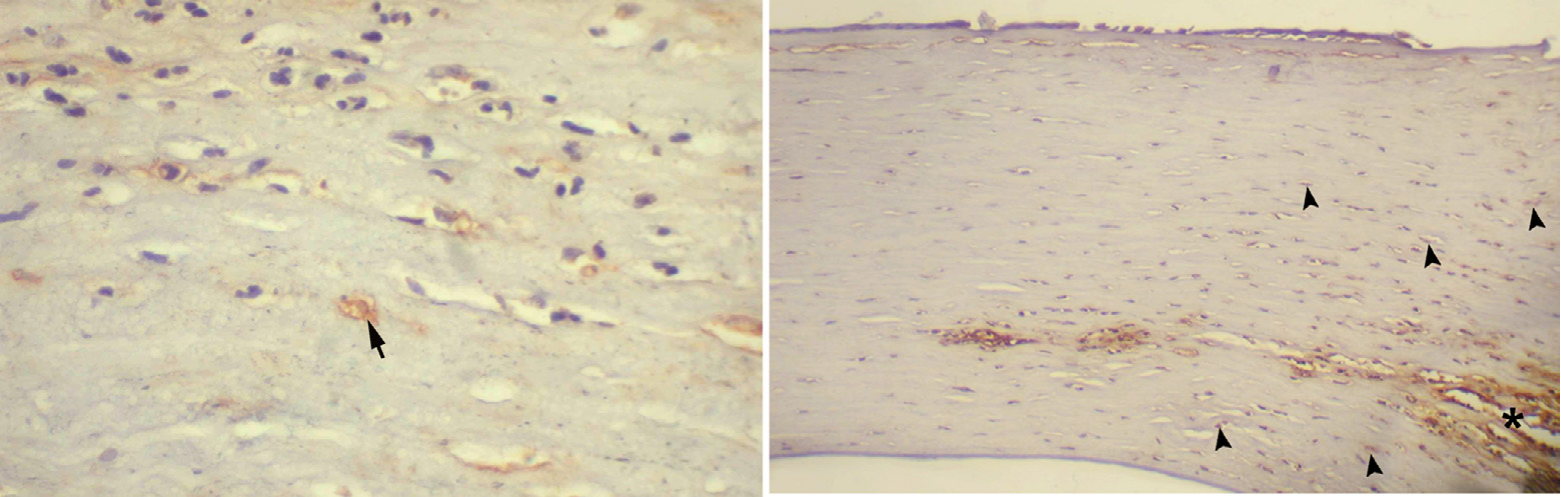
The corneal stroma in a case of fungal keratitis shows diffuse TUNEL positivity in the zone of inflammation (*) as well as discrete positive staining in the peripheral keratocytes away from zone of inflammation (arrow head) (TUNEL, ×250) [Left]. The higher magnification of the same shows TUNEL positive nuclei of the keratocyte (Arrow) in a clean background, free of inflammatory cells. (TUNEL staining, ×500) (TUNEL ×400) [Right]

**Figure 5:**
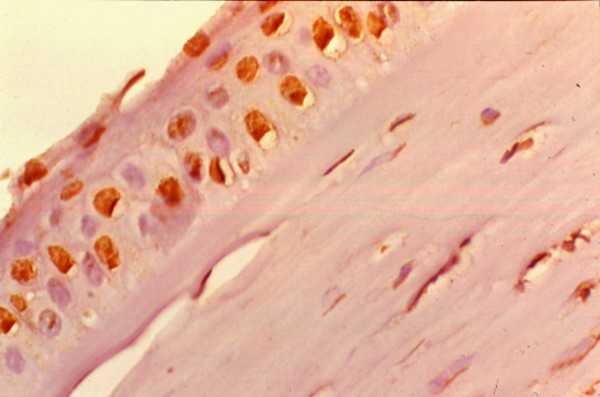
Section from a case of viral keratitis showing TUNEL staining in the epithelial cells. The underlying stroma shows inflammatory cells and irregular keratocytic nuclei. (TUNEL, ×500)

TUNEL staining could be assessed in 53 cases, rest 6 cases, it could not be evaluated due to either a total loss of keratocytic nuclei in the stroma, or absence of inflammation-free zone of corneal stroma. The TUNEL positive brown cells in the stromal region were noted in the region of inflammation with necrosis as well as in the surrounding stroma (figure 3), away from the zone of inflammation. In the zone of inflammation, the TUNEL positivity was seen as diffuse staining of the background as well as within the neurtrophils seen in that region. In the surrounding stroma, the TUNEL positivity was seen within the keratocytic nuclei as linear or granular nuclear staining (figure 4). This was seen in 53 (90%) cases, located mostly in the deep stroma (29; 49%) and in few cases the peripheral stroma (20; 34%). It was seen in 19 of 22 cases of fungal, 14/14 cases of bacterial, 14 of 15 cases viral and 6 of 8 cases of *Acanthamoeba *etiology. In addition, TUNEL positive cells were seen in the epithelium in 10 cases, (figure 5) located in the basal layer, middle layers as well as the superficial cells. Normal nuclei were also noted. The control slides showed no positive staining within the corneal stroma. Occasional positive cells were noted in the superficial cells of the corneal epithelium.

The keratocyte changes and the TUNEL staining was compared to the size of ulcer, thinning, degree of inflammation, necrosis and etiologic agent, as shown in Table 3. The correlation between apoptotic cell death (TUNEL positive cells) and the various types of infections could not be evaluated in view of high prevalence of positive staining in all types of infection. The epithelial cell apoptosis was seen in 10 cases, of which 6 were of viral etiology.

## Discussion

Inflammation of the avascular transparent cornea is unique and different from other tissues in the body. Breakdown of the physiological barrier of epithelium and the tear film leads to the entry of microorganisms, which proliferate in the stroma. This is followed by a sequence of events including edema, inflammatory cell influx from tears, limbus, abscess formation and necrosis of the stroma [2]. When healing initiates there is epithelial migration of epithelial cells into the crater of the ulcer, along with growth of blood vessels into the stroma, proliferation and migration of stromal fibroblasts and influx of macrophages for scavenger activity, finally resulting in the scar formation. The degree of opacification and visual dysfunction appears to be related to the number and density of inflammatory cells and their location, and to the presence or absence of associated enzymatically induced stromal damage, vascularization, and other secondary changes. The visual recovery would therefore depend on the elimination of the inflammatory cell infiltrates, regression of edema, scarring and the newly developed vessels.

Though the observation that the stromal keratocytes disappear after epithelial injury was published more than 30 years ago, the nature of cell death was attributed to apoptosis by Campos et al and subsequently by Wilson SE et al [16-18]. Wilson et al also reported keratocyte apoptosis in association with viral infection of the overlying corneal epithelium by the herpes simplex virus [19]. They had hypothesized that one of the primary functions of keratocyte apoptotic response is to inhibit the virus that infects the corneal epithelium from spreading into the stroma and to deeper areas by temporarily eliminating the cells that are needed for viral replication and extension. This is in accordance to the Samurai principle in biology " it is better to die than do a wrong", where the damaged cells commit suicide or the programmed cell death by apoptosis [20,21].

Apoptotic cell death can be evaluated by many tests like DNA laddering as documented by gel electrophoresis, detection of enzymes like caspases, expression of mRNA of the corresponding proteins and enzymes involved in the process and by TUNEL staining [22-26]. The latter involves labeling the nick ends of the DNA fragments by labeled digitoxin molecules mediated by tdt transferase enzyme. The advantage of this technique is it identifies the apoptotic cell death in the formalin-fixed, paraffin embedded tissues and thus can be interpreted in the light of other morphological changes that take place in the stromal tissues in infections of cornea.

Ours is the first study demonstrating apoptosis in corneal tissue obtained from patients diagnosed as fungal, bacterial, viral and *Acanthamoeba *infections. A few animal experiments document the phenomenon of apoptosis in inflammatory conditions. In an animal experiment using rabbits, Moreau et al demonstrated that intrastromal injection of purified alpha-toxin mediates cell death of epithelial cells both by necrosis and apoptosis [27]. Similar observations have been made in our study where the infected corneal tissues showed both necrosis and apoptotic cell death. The diffuse TUNEL positive staining in the region of inflammation and necrosis also supports the hypothesis that the inflammatory cells also undergo apoptotic cell death. This process could be self-induced or due to the inflammatory cytokines which are capable of inducing the apoptosis of the host cells and also triggering apoptosis in themselves. Similar kind of observation has been made by Ozaki et al in artificially induced Arthus reaction, wherein they demonstrated that the inflammatory cells and the new blood vessels formed in the cornea regress by the process of apoptosis thereby reducing the haze and leading to corneal transparency [28]. A few other reports show that PRK produces more haze than lasik, not only because of the lack of the epithelial covering above but because of the polymorphonuclear infiltrates [29]. Therefore it could be speculated that apoptotic cell death is more of a protective phenomenon and reduces the degree of scarring that could result from necrosis alone. How this would affect the healing process and the scarring following the control of infection in early stages of corneal infection in humans in another aspect which requires further studies. It is not the scope of this study to look for the mediators of apoptosis, but evidence in literature points to the role of Fas-Fas ligand, interleukin 1, enzymes and free radicals in this process [30,31].

Loss of keratocytic nuclei was detected in 73% of cases on light microscopy, while TUNEL stain detected apoptotic cell death in 83% of cases included in the study. In 6 cases, it could not be assessed due to either the total loss of keratocytes, or due to presence of inflammatory infiltrates in all regions of corneal stromal, without an inflammatory-free zone for proper interpretation. This makes us suspect that probably it is seen in all cases of infectious keratitis. The keratocyte loss was noted mostly in the deep posterior stroma. This is similar to our study on *Acanthamoeba *keratitis where the remaining keratocytes in the posterior stroma showed TUNEL positivity [12]. The keratocyte loss following epithelial scraping is noted in the superificial stroma, which is the first region to be exposed following the insult to the epithelium [32]. In tissues from corneal ulcer as used in this study, the injurious stimulus is present both in the epithelium and the superficial stroma, the deeper keratocytes are possibly are continuously exposed to the insult, thus initiating the suicidal cell death. Since this study includes only those cases of infectious keratitis which underwent therapeutic keratoplasty and whose buttons histologically showed evidence of preserved stroma to comment upon, it is difficult to extrapolate the sequence of events in different phases of the infective process. Planned animal experiments could possibly throw more light on these events. But with the knowledge that the apoptotic bodies are rapidly removed form the tissues by the neighboring cells and the scavenger cells, it is possible for us to presume that apoptotic cell death does occur in late phases of the infectious process. Though TUNEL assay is an accepted method of morphological detection of apoptosis in cells and tissues, assessment of apoptosis by an additional method would have been more informative and could have overcome the limitation of this study.

## Conclusion

In summary, the corneal tissues involved by various types of infection including fungal, viral and bacterial infections show evidence of inflammation, necrosis as well as apoptotic cell death of keratocytes and inflammatory cells. We speculate that the keratocyte loss adjacent to the infected tissue could be a protective phenomenon, which requires evaluation by further studies.

## Competing interests

The author(s) declare that they have no competing interests.

## Authors' contribution

This work was conceived and planned by Geeta K Vemuganti. Clinical and microbiological support was provided by Prashant Garg and Savitri Sharma respectively. The bench work and analysis was done by Kishore Reddy and Ghazala Iftekhar.

## Pre-publication history

The pre-publication history for this paper can be accessed here:



## Figures and Tables

**Table 1:** Clinical features of cases at presentation.

Clinical features		Frequency(%)
Size of ulcer * (38)	Small	6 (15.8%)
	Medium	15 (39.5%)
	Large	17 (44.7 %)
Location of ulcer* (42)	Central	28 (66.7%)
	Peripheral	6 (14.3%)
	Total	8 (19.0%)
Depth of infiltrates* (38)	Anterior	4 (10.5 %)
	Middle	15 (39.5%)
	Posterior	19 (50.0%)
Perforation (43)	Absent	40 (93.0%)
	Present	3 (7.0%)
Thinning (43)	Absent	24 (55.8%)
	Present	19 (44.2%)
Infiltrates in Anterior chamber*(34)	Absent	4 (11.8%)
	Present	30 (88.2%)

• Due to missing data valid percentage has been considered

**Table 2:** Histologic features of excised corneal tissue of patients with infectious keratitis

Histologic changes		Frequency of histologic changes in 59 cases
Epithelial Ulceration	Absent	7 (11.9%)
	Present	52 (88.1%)
Bowmans membrane	Absent	58 (98.3%)
	Present	1 (1.7%)
Inflammation	Mild/Mod	28 (47.5%)
	Severe	31 (52.5%)
Vascular channels	Absent	51 (86.4%)
	Present	8 (13.6%)
Stromal necrosis	Absent	11 (18.6%)
	Present	48 (81.4%)
Keratocyte loss*	Absent	8 (13.6%)
	Reduced	47 (79.7%)

• could not assessed in two cases

**Table 3:** Comparison of clinical, histologic features and degree of keratocyte loss in various types of corneal infections

Features		Etiology				Correlation of apoptosis with clin/hist features
	
		Fungus (22)	Virus (15)	Bacteria (14)	Acanth (8)	
Size of ulcer (38)	Small (≤2 mm^2^)	1 (6.7%)	2 (28.6%)	1 (9.1%)	2 (40.0%)	P = 0.224
	Medium and Large (≥25 mm^2^)	14 (63.63%)	5 (33.33%)	10 (71.4%)	3 (37.5%)	
Stromal thinning (43)	Absent	10 (62.5%)	3 (42.9%)	6 (42.8%)	5 (83.3%)	P = 0.313
	Present	6 (37.5%)	4 (57.1%)	8 (57.1%)	1 (16.7%)	
Necrosis (59)	Absent	0	8 (53.3%)	2 (14.3%)	1 (12.5%)	NA
	Present	22 (100%)	7 (46.7%)	12 (85.7%)	7 (87.5%)	NA
Inflammation (59)	Mild/Moderate	6 (27.3%)	10 (66.7%)	7 (50.0%)	5 (62.5%)	P = 0.087
	Severe	16 (72.7%)	5 (33.3%)	7 (50.0%)	3 (37.5%)	
Keratocytes (57)	Absent	4 (19.1%)	4 (28.6%)	0	0	NA
	Reduced	15 (71.4%)	10 (71.4%)	14 (100%)	8 (100%)	
	Normal	2(9.5%)	0	0	0	
Apoptosis (54)	Absent	0	1 (6.7%)	0	0	NA
	Present	19 (100%)	14 (93.3%)	14 (100%)	6 (100%)	

NA = Not applicable
